# Male lifespan extension with 17‐α estradiol is linked to a sex‐specific metabolomic response modulated by gonadal hormones in mice

**DOI:** 10.1111/acel.12786

**Published:** 2018-05-27

**Authors:** Michael Garratt, Kim A. Lagerborg, Yi‐Miau Tsai, Andrzej Galecki, Mohit Jain, Richard A. Miller

**Affiliations:** ^1^ Department of Pathology University of Michigan Medical School Ann Arbor Michigan; ^2^ Departments of Medicine & Pharmacology University of California San Diego San Diego California; ^3^ University of Michigan Geriatrics Center Ann Arbor Michigan

**Keywords:** ageing, estrogen, gender, life‐extension, metabolomics, testosterone

## Abstract

Longevity in mammals is influenced by sex, and lifespan extension in response to anti‐aging interventions is often sex‐specific, although the mechanisms underlying these sexual dimorphisms are largely unknown. Treatment of mice with 17‐α estradiol (17aE2) results in sex‐specific lifespan extension, with an increase in median survival in males of 19% and no survival effect in females. Given the links between lifespan extension and metabolism, we performed untargeted metabolomics analysis of liver, skeletal muscle and plasma from male and female mice treated with 17aE2 for eight months. We find that 17aE2 generates distinct sex‐specific changes in the metabolomic profile of liver and plasma. In males, 17aE2 treatment raised the abundance of several amino acids in the liver, and this was further associated with elevations in metabolites involved in urea cycling, suggesting altered amino acid metabolism. In females, amino acids and urea cycling metabolites were unaffected by 17aE2. 17aE2 also results in male‐specific elevations in a second estrogenic steroid—estriol‐3‐sulfate—suggesting different metabolism of this drug in males and females. To understand the underlying endocrine causes for these sexual dimorphisms, we castrated males and ovariectomized females prior to 17aE2 treatment, and found that virtually all the male‐specific metabolite responses to 17aE2 are inhibited or reduced by male castration. These results suggest novel metabolic pathways linked to male‐specific lifespan extension and show that the male‐specific metabolomic response to 17aE2 depends on the production of testicular hormones in adult life.

## 1 INTRODUCTION

Males and females in most species differ in their aging rates and lifespan. In humans, sexual dimorphism is observed in the onset, severity, and/or frequency of age‐associated metabolic dysfunction, frailty, cancers, and some forms of neurological disease, with part of this disparity linked to the underlying effects of sex‐specific gonadal hormone production across life (Legato, 2010). Male testicular production of testosterone has been postulated to reduce male lifespan compared to females (Brooks & Garratt, 2017), and castration has been associated with longer lifespan in several mammal species, including humans (Hamilton & Mestler, 1969; Min, Lee & Park, 2012), primates (Kessler et al., 2016), sheep (Jewell, 1997), and rodents (Asdell, Doornenbal, Joshi & Sperling, 1967; Muehlbock, 1959), suggesting a potential common and conserved biological mechanism. At the same time, estrogen production has been linked to female‐specific health benefits (Regan & Partridge, 2013), with ovariectomy reported to reduce female rodent lifespan in some instances (Benedusi et al., 2015; Mason, Cargill, Anderson & Carey, 2009).

17‐β estradiol is the dominant female sex hormone produced from the ovaries in adult life. Female‐specific production of 17‐β estradiol contributes to sex differences in metabolism, supported by the observation that circulating 17‐β estradiol production declines during menopause, a fall associated with lower glucose homeostasis and elevated visceral adiposity (Mauvais‐Jarvis, 2015). However, other estrogens, and estrogenic actions outside of the classical ER receptors, are increasingly recognized to have potential health and anti‐aging benefits, which may mimic or operate outside of the effects of 17‐β estradiol on its best‐characterized receptors. This study focuses on a stereoisomer of 17‐β estradiol, 17‐α estradiol (17aE2). Due to the stereochemistry of the carbon atom 17, 17aE2 has a much weaker binding affinity to the classical estrogen receptors, and in some situations has a greater binding affinity for other estrogen receptors, including the brain ER receptor ER‐X (Toran‐Allerand, Tinnikov, Singh & Nethrapalli, 2005; Toran‐Allerand et al., 2002). 17aE2 has a range of bioactive properties, including inflammatory and antioxidant effects (Moos, Dykens, Nohynek, Rubinchik & Howell, 2009), and an ability to inhibit the activity of 5‐alpha reductase enzymes (Schriefers, Wright, Rozman & Hevert, 1991), which convert testosterone to dihydrotestosterone, a more potent activator of the androgen receptor. Treatment of male mice with 17aE2 has been shown to extend median lifespan by 19% (Strong et al., 2016), ameliorate age‐associated metabolic and inflammatory dysfunction (Stout et al., 2016), and improve male glucose tolerance across much of adult life (Garratt, Bower, Garcia & Miller, 2017).

The effects of 17aE2 on lifespan and metabolic health are strongly sex‐specific. Females accrue no detectable metabolic benefit of 17aE2 treatment, and no life extension has been observed at the two tested concentrations to date (Harrison et al., 2014; Strong et al., 2016). We have previously observed that male‐specific increases in glucose tolerance and hepatic mTORC2 signaling with 17aE2 treatment are inhibited in males that are castrated in adulthood, prior to treatment onset (Garratt et al., 2017). Ovariectomy (OVX) of females prior to treatment with 17aE2 had little effect on these physiological responses, with OVX females showing no improvement in glucose tolerance after 17aE2 treatment. The modulation of sex‐specific responsiveness to 17aE2 with male castration, but not female ovariectomy, suggests that the sex‐specificity in responsiveness to 17aE2, at least in relation to glucose tolerance, is caused by an interaction with male gonadal hormones, such as production of testosterone.

Untargeted metabolomics provides a powerful approach to understand the underlying metabolic responses that occur after drug treatment or other manipulations. Association of metabolite changes with particular pathways can reveal the biochemical and metabolic processes consistently altered by an intervention, and molecules that are identified as most strongly affected by an intervention are candidates for a role in the intervention response. In this study, we used untargeted metabolomic analysis of liver tissue to identify metabolite responses that occur in mice treated with 17aE2 at the concentration previously shown to extend male but not female lifespan. The liver is a key tissue involved in regulation of glucose, carbohydrate, and protein metabolism, with previous research showing that the metabolite profile of the liver changes in response to lifespan extending manipulations in line with expected alterations in energy utilization (e.g., calorie restriction (Green et al., 2017)). We focused our investigation on liver metabolites that show a sex‐specific response to 17aE2 and may underlie the male‐specific lifespan response. Comprehensive metabolomics revealed previously unrecognized metabolite changes that were male‐specific in response to 17aE2 and were abrogated by male castration. These studies suggest that sex‐specific metabolite responses to 17aE2 are modulated by testicular hormones and provide new insight into the mechanisms of sexual dimorphism in lifespan extension.

## 2 RESULTS

### 2.1 Untargeted assessment of sex‐specific metabolite responses to 17aE2

To uncover the metabolic underpinnings of sex‐specific lifespan extension with 17aE2, male and female mice were treated with 17aE2 for eight months starting at 4 months of age, after which liver samples were subjected to comprehensive untargeted metabolomics analysis of polar and nonpolar (lipid) metabolites. Liquid chromatography–mass spectrometry (LC‐MS)‐based metabolomics detected 10,271 unique spectral features in liver. To identify metabolites that show a change with treatment consistent with lifespan extension (altered in males but not females), we ran two‐way ANOVAs for each metabolite, including sex, treatment, and an interaction between sex and treatment as factors. We focused on the interaction as the statistical term of interest, and prioritized metabolites that were altered in males but not females, as these changes were correlated with the male‐specific lifespan response.

**Figure 1:**
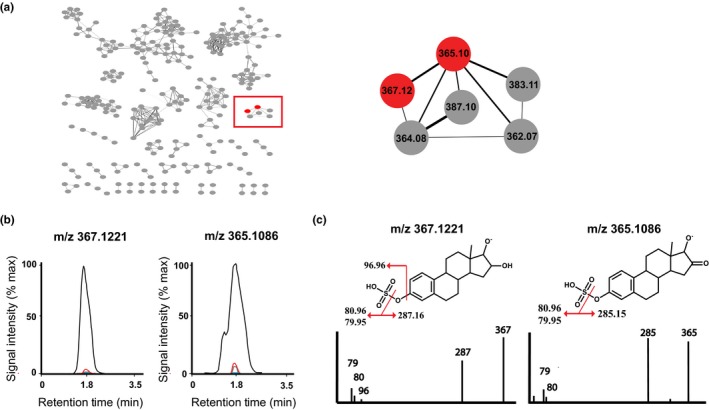
Identification of two metabolites that show a sex‐specific response to 17aE2. (a) Chemical networking of unknown metabolites, with nodes representing an individual metabolite MS/MS spectrum and edge thickness, indicating cosine similarity between metabolites. Area within the red box is highlighted with red nodes representing two related metabolites at m/z 367.1221 and 365.1086 from among the four prioritized metabolites. (b) Chromatograms for metabolites m/z 367.1221 and m/z 365.1086 in the four animal groups, with male control group (blue), female control group (gray), male 17‐α estradiol group (black), and female 17‐α estradiol group (red). (c) MS/MS, structure, and fragmentation markup for metabolites m/z 367.1221 and m/z 365.1086, identifying the metabolites as estriol 3‐sulfate and 16‐oxoestradiol 3‐sulfate, respectively

Across the initial >10,000 spectral features, we identified six metabolites that showed a significant sex by treatment interaction after correction for multiple comparisons (FDR *p *< 0.05; Table 1). Four of these showed a change primarily in males but not females, of which one was strongly reduced in livers of 17aE2‐treated males, while three were strongly increased in males. These metabolites represent sex‐specific signals that correlate with the sex‐specific lifespan response (and could be used to determine the effects of gonadectomy on treatment responses, see below). Chemical networking of the four prioritized metabolites revealed two metabolites at m/z 367.1221 and m/z 365.1086 to be chemically related, with distinct clustering (Figure 1a). Manual inspection of extracted ion chromatograms for both related peaks confirmed a stronger elevation in males treated with 17aE2 than in females (Figure 1b). Manual inspection of fragmentation MS/MS spectra for the two unknown metabolites at m/z 367.1221 and m/z 365.1086 revealed them to be estriol‐3‐sulfate and 16‐oxoestradiol 3‐sulfate, respectively (Figure 1c). Estriol‐3‐sulfate and 16‐oxoestradiol 3‐sulfate are two estrogenic compounds of a similar chemical structure and may be metabolized from 17aE2 via 16 alpha hydroxylation (Longcope 1984). These results indicate that 17aE2 undergoes sex‐specific metabolism in male mice to secondary estrogenic products.

**Table 1:** Metabolites showing a sex‐specific response to 17aE2 treatment. Data are split into whether the metabolite was initially untargeted or was identified by standard during analysis. M.Z. and retention time (RT, minutes) are shown for untargeted metabolites. Analysis is then further split by whether the metabolite change is seen in females or males. *p*‐values shown in Table 1 are uncorrected for multiple comparisons

Metabolite			Treatment interaction (*p*‐value two‐Way ANOVA)			Effect of 17aE2 (*p*‐value Student's *t* test)			
			Sex (intact mice)	Cast (male)	OVX (female)	Male	Female	Cast Male	OVX Female
Sample size control						8	7	8	8
Sample size 17aE2						9	8	8	8
Untargeted metabolites									
Male response	M.Z.	R.T.							
MET01834	461.2	1.49	0.000014	0.0050	0.22	↑ 0.000001	0.97	↑ 0.0000	0.14
Estriol‐3‐sulfate	367.1	1.80	0.000017	0.0000	0.23	↑ 0.000013	0.16	↑ 0.0012	0.15
MET03323	437.0	2.75	0.000027	0.0000	0.005	↓ 0.000006	0.82	0.39	0.0059
16‐oxoestradiol 3‐sulfate	365.1	1.78	0.000029	0.0060	0.16	↑ 0.000007	0.12	↑ 0.0008	0.13
Female response									
MET03545	377.3	3.45	0.000001	0.31	0.0000	0.38	↓ 0.0000	0.32	0.058
MET03666	459.3	3.45	0.000001	0.29	0.0000	0.77	↓ 0.0000	0.33	0.057
Identified by standard									
Male response									
Phosphocholine			0.00074	0.23	0.51	↑ 0.0010	0.13	0.37	0.79
4‐imidazoleacetic acid			0.0012	0.02	0.14	↑ 0.0017	0.29	↑ 0.029	0.25
N‐alpha acetyl_Lysine			0.0061	0.018	0.61	↑ 0.0040	0.62	0.67	↓ 0.048
n‐alpha‐acetyl‐l‐asparagine			0.00998	0.063	0.37	↑ 0.024	0.18	0.78	0.57
Female response									
Betaine			0.00077	0.062	0.065	0.13	↓ 0.0019	0.27	↓ 0.044
Changed in both									
n‐acetyl‐dl‐glutamic acid			0.0076	0.13	0.082	0.081	↓ 0.016	0.58	0.91
l‐serine			0.0083	0.038	0.19	0.056	0.072	0.61	0.085
Corticosterone			0.0084	0.0070	0.33	↑ 0.039	0.067	0.089	0.59

### 2.2 Modulation of liver metabolome with 17aE2 treatment

To test whether 17aE2 results in coordinated changes in metabolites associated with specific metabolic processes, a subset of metabolites were identified from untargeted metabolomic analysis of liver samples following treatment with 17aE2. Using pure standards, 148 metabolites were identified based on retention time, accurate mass characteristics, and MS/MS fragmentation patterns. Twenty‐five of the 148 metabolites were significantly altered in abundance in the livers of 17aE2‐treated males compared to untreated males (uncorrected for multiple comparisons), with the vast majority of metabolites (24/25) found to increase (Table 2). 17aE2 was found to result in a similar (24) number of metabolite changes in female livers (Table S1), although only three fatty acids—eicosatrienoic acid, docosatrienoic acid, and behenic acid—increased in the livers of both sexes with treatment. Approximately 50% of the metabolites significantly altered in abundance in 17aE2‐treated females decrease in abundance compared to controls.

**Table 2:** Metabolites that are significantly affected by 17aE2 treatment in males. Arrows indicate direction of change with 17aE2 treatment. *p*‐values are generated by a Student's t test, with the exception of data for arginine, where a Mann–Whitney U test was used because of the presence of several zero values. The comparable analysis for each metabolite is also shown for females, castrated males, and OVX females. Sample sizes are shown in Table 1

ID	Male		Female		Castrated males		OVX females	
	Change	*p* value	Change	*p* value	Change	*p* value	Change	*p* value
Phosphocholine	↑	0.001		0.130		0.368		0.789
4‐Imidazoleacetic acid	↑	0.002		0.291	↑	0.029		0.253
Docosahexaenoic Acid	↑	0.003		0.491		0.827		0.919
Arginine	↑	0.004		0.460		0.560		0.160
N alpha‐acetyl‐L‐lysine	↑	0.004		0.624		0.671	↓	0.048
Docosadienoic Acid	↑	0.006		0.211		0.597		0.098
Allothreonine	↑	0.006		0.708		0.747		0.977
Glutamine	↑	0.006		0.128		0.212		0.124
Eicosatrienoic Acid	↑	0.010	↑	0.035		0.535	↑	0.047
Ornithine	↑	0.012		0.594		0.969		0.457
Adrenic Acid	↑	0.013		0.744		0.575		0.131
Eicosadienoic Acid	↑	0.015		0.118		0.746		0.202
Argininosuccinic acid	↑	0.015		0.826		0.598		0.791
Alanine	↑	0.016		0.093		0.945		0.025
D‐Ribulose 5‐phosphate	↑	0.020		0.224		0.395	↑	0.778
Behenic Acid	↑	0.024	↑	0.014		0.671		0.690
N‐alpha‐acetyl‐L asparagine	↑	0.024		0.175		0.785		0.569
Valine	↑	0.027		0.772		0.923		0.790
Proline	↑	0.029		0.211		0.559		0.346
Docosatrienoic Acid	↑	0.032	↑	0.018		0.737		0.264
N‐aceytl‐DL‐serine	↑	0.037		0.273	↑	0.004	↑	0.021
Arachidonic Acid	↑	0.037		0.666		0.885		0.901
Dethiobiotin	↑	0.038		0.600		0.126		0.521
Corticosterone	↑	0.039		0.067		0.089		0.591
Trans‐4‐hydroxyproline	↓	0.049		0.455		0.442		0.921

We conducted Quantitative Metabolomic Set Enrichment Analysis to test whether 17aE2 leads to coordinated shifts in metabolites associated with any particular metabolic process. This analysis uses generalized linear models to estimate the association between concentration profiles of different metabolites linked to a particular metabolic process and can detect subtle changes in coordinated sets of metabolites (Xia & Wishart, 2010). We conducted this analysis separately for each sex, as we were particularly interested in metabolites and metabolomic responses that changed specifically in males, and thus are associated with male‐specific lifespan extension. Metabolite responses in each sex were referenced against the “metabolite pathway database,” which links groups of metabolites to 88 different pathways. Metabolite pathway associations with 17aE2 treatment within each sex are shown in Table S2. For males, 17aE2 treatment leads to a significant shift in metabolites enriched in pathways associated with “protein biosynthesis” and the “urea cycle.” By contrast, for females, 17aE2 treatment leads to modulation of metabolites associated with “betaine metabolism.” This mainly reflects the effect of 17aE2 treatment in females reducing hepatic levels of betaine and dimethylglycine, and increasing S‐adenosylhomocysteine (Fig. S1).

**Figure 2:**
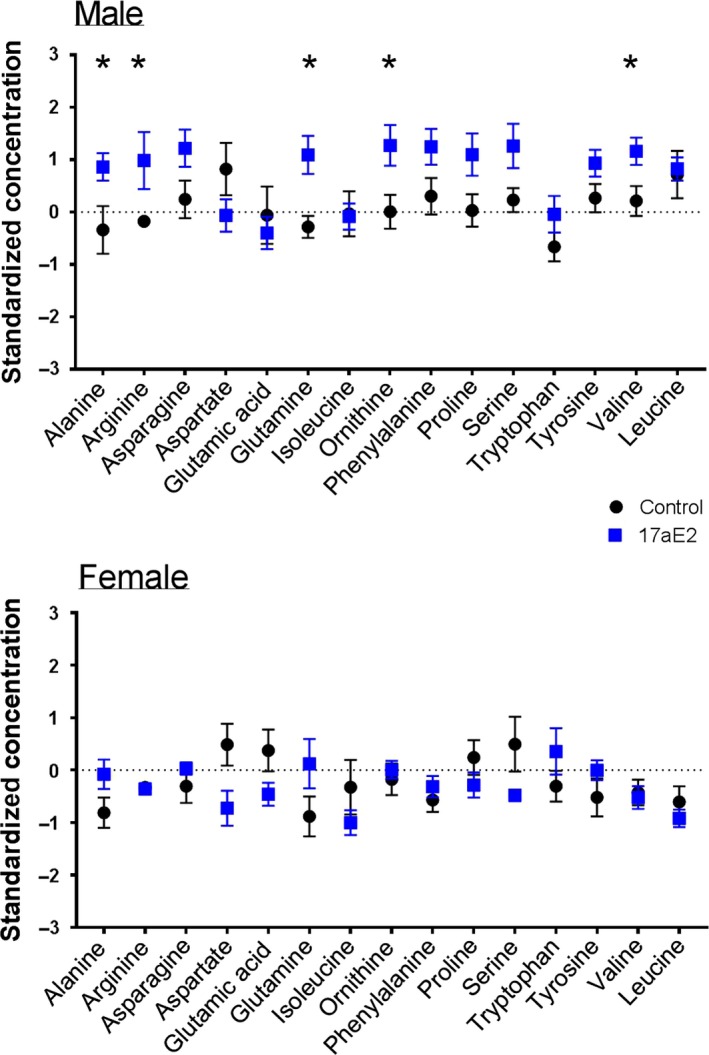
Elevated hepatic amino acids in males treated with 17aE2. Enrichment analysis (Table S2) showed that metabolites associated with protein biosynthesis were significantly enriched in males treated with 17aE2. This reflects an increase in the abundance of a range of amino acids, an effect that was observed in males but not females. * = *p* < 0.05 calculated with a Student's *t* test. *N *= 7–9 per treatment group, per sex. Error bars show mean ± SEM for standardized abundance values

**Figure 3:**
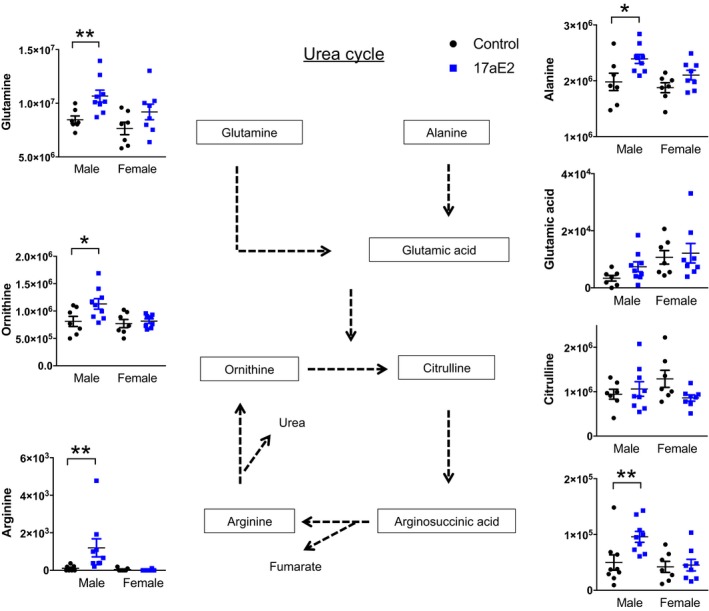
Changes in metabolites associated with the urea cycle in livers of 17aE2‐treated mice. Enrichment analysis showed that males treated with 17aE2 show a significant enrichment in metabolites associated with the urea cycle, while these effects are not observed in 17aE2‐treated females. * = *p* < 0.05, **p < 0.01 calculated with a Student's t test. *N *= 7–9 per treatment group, per sex. Error bars show mean ± SEM, and individual data points show the intensity value for each individual

In males, pathway enrichment for metabolites associated with “protein biosynthesis” reflected the induction by 17aE2 of a male‐specific elevation in many amino acids, including alanine, arginine, glutamine, ornithine, and valine (Figure 2). Metabolite enrichment for the urea cycle reflected increases in both alanine and glutamate, and direct urea cycle intermediates, including argininosuccinic acid, arginine, and ornithine (Figure 3).

### 2.3 Identified metabolites showing a sex‐specific response to 17aE2

**Figure 4:**
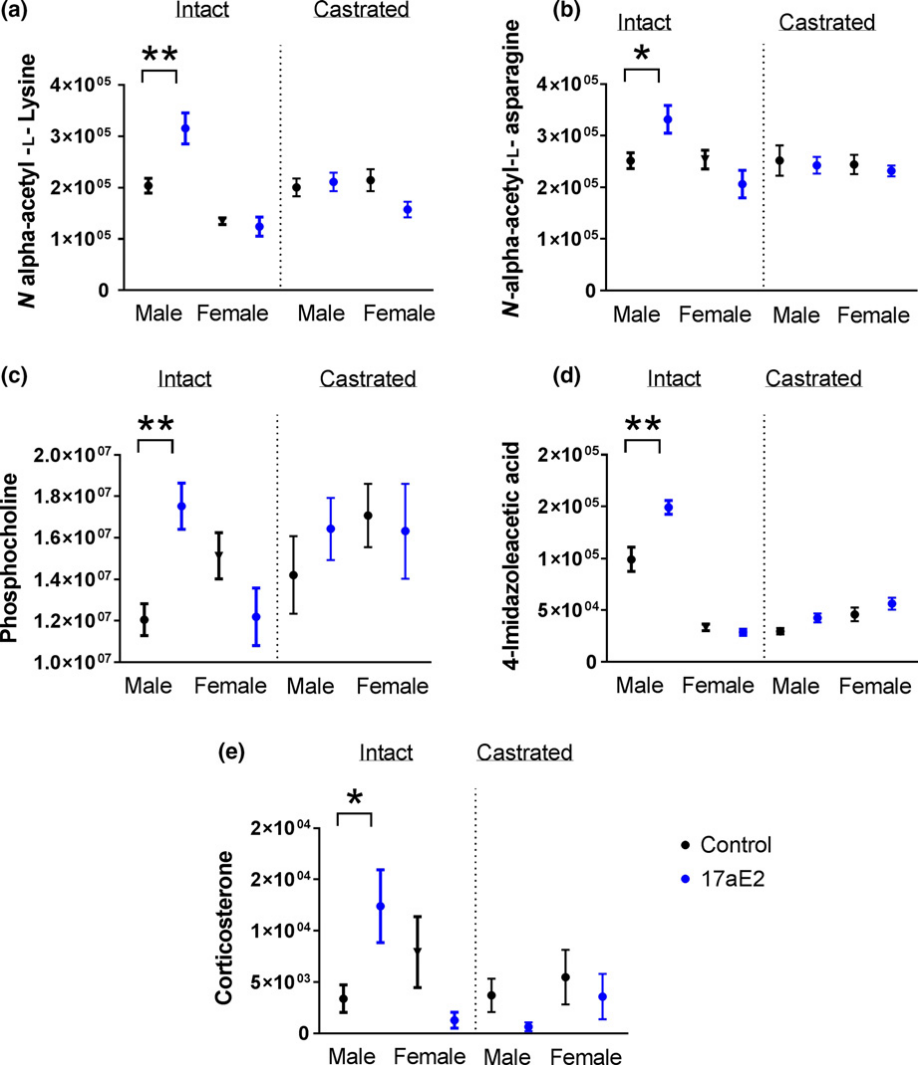
Identified metabolites showing a sex‐specific response to 17aE2 treatment and a change in treated males, and inhibition of these sex‐specific responses by male castration. Statistics are shown in Table 1. * = *p* < 0.05, **p < 0.01 calculated with a Student's t test. *N *= 7–9 per treatment group, per sex. Error bars show mean ± SEM for abundance values of each metabolite

We further assessed changes in individual known metabolites with 17aE2 treatment, again focusing on metabolites that change specifically in males but not females, as indicated by a significant two‐way interaction between sex and treatment, and also showing a nominally significant response to 17aE2 in males. Table 1 shows those known metabolites that show a differential response to 17aE2 treatment according to sex; the associated *p*‐values are shown in the “Sex” column for intact mice. Most notable is the male‐specific increase in N‐acetylated amino acids, lysine and asparagine (Figure 4a,b). N‐acetylated amino acids, including N‐acetyl lysine, are strongly correlated to urea cycle metabolite concentrations in human red blood cells collected from old (>80 years) humans (Chaleckis, Murakami, Takada, Kondoh & Yanagida, 2016). While these are increased by 17aE2 in liver of males they are unaffected in females (Figure 3). The metabolite showing the most significant sex*17aE2 interaction within the known metabolite dataset is phosphocholine (Sex*Treatment interaction: raw *p*‐value <0.0007; Table 1; Figure 4c), which is increased in males but unaffected in females, and can be produced as a product of serine, glycine and threonine metabolism. Also of interest, and potentially linked to protein metabolism, is the male‐specific increase in corticosterone, which in females decreases, though not significantly (Figure 4E; Table 1). We have previously shown that activation of the serum glucocorticoid receptor 1 (SGK1), a direct target of corticosterone, is also elevated sex‐specifically in 17aE2‐treated males (Garratt et al., 2017). The sex‐specific metabolite that represents a change specifically in females is betaine, which is decreased in 17aE2‐treated females compared to controls (Table 1; Fig. S1).

### 2.4 Sex‐specific metabolomic responses are inhibited by male castration

**Figure 5:**
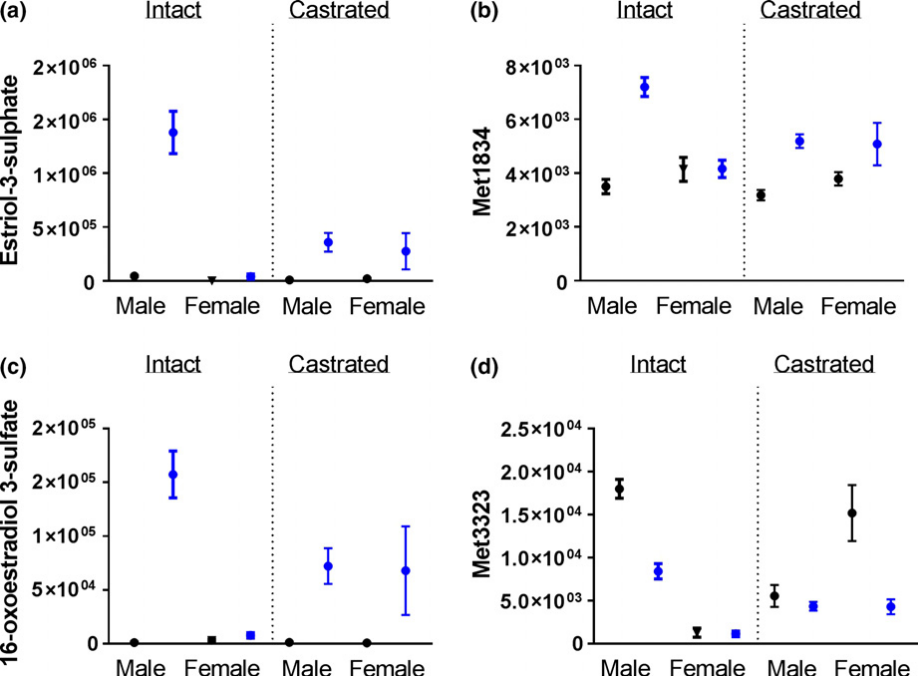
Metabolites showing a sex‐specific response to 17aE2 treatment in intact animals. Figure shows the metabolites that were subsequently identified as estriol‐3 sulfate (a) and 16‐oxoestradiol 3‐sulfate (c). In each panel, data are shown for 17aE2‐treated intact animals on the left and animals that had gonads removed prior to treatment on the right. *N *= 7–9 per group. Statistics are shown in Table 1. Error bars show mean ± SEM

To test whether the sex‐specific metabolomic effects of 17aE2 are generated by the underlying effects of male or female gonadal hormones, we castrated male mice and ovariectomized female mice at three months of age, that is, one month prior to administration of 17aE2, and tested whether this postpubertal gonadectomy altered sex‐specific metabolite responses to 17aE2. These animals were produced and evaluated in parallel with the intact (sham‐operated) animals described above. All six of the metabolites showing a sex by treatment interaction (i.e., those where FDR < 0.05) across the untargeted dataset showed a response that was dependent on gonadectomy. The four metabolites strongly altered in 17aE2‐treated male livers, which include estriol‐3‐sulfate and 16‐oxoestradiol 3‐sulfate, show a significantly diminished response to 17aE2 treatment in castrated males (Figure 5), indicated by the interaction terms within males for “Castration” in Table 1.

We also tested whether gonadectomy altered the effects of 17aE2 treatment on known metabolites associated with specific metabolic pathways in either sex. Of the 25 known metabolites altered by 17aE2 treatment in normal (sham)‐treated males, only two (4‐imidazoleacetic acid and N‐acetyl‐DL‐serine) were also significantly changed, in the same direction, in castrated males treated with 17aE2 (Table 2). For 4‐imidazoleacetic acid the response in this metabolite with 17aE2 was significantly diminished, as indicated by the “castration” interaction in Table 1. When considering known metabolite responses to 17aE2 in castrated males, enrichment analysis failed to show a significant association in metabolite responses with any particular biochemical pathway after correction for FDR, and there was no evidence that amino acids or metabolites of the urea cycle were affected by 17aE2 treatment in castrated males (Table S3; Fig. S2). Thus, the male‐specific elevations in metabolites associated with protein biosynthesis and the urea cycle with 17aE2 treatment require male‐specific production of gonadal hormones. Ovariectomized females did not show significant enrichment for any specific biochemical pathway after correction for FDR, although it is notable that betaine metabolism, which is altered in sham females, was also modulated in OVX females in the analysis before correction for multiple comparisons by the FDR calculation, and this pathway did produce FDR = 0.07 in the ovariectomized females (Table S3).

### 2.5 Sex‐specific metabolite responses in plasma are also inhibited by castration

To test whether the testosterone dependence of male‐specific responses is an effect that is restricted to the liver, or represents a more general sexual dimorphism in metabolomic response, we assessed metabolomic responses to 17aE2 treatment in plasma. As the strongest sex‐specific responses in liver were observed for bioactive lipids, we restricted our analysis to this sampling method. We observed 13 metabolites that both showed a significant sex*17aE2 interaction after correction for FDR and represented responses where the metabolite changed significantly in intact males but not females (thus correlating with the lifespan response). In each case, the significant male response was not observed in castrated males (Table S4), highlighting its dependence on male gonadal hormones, just as in the analysis of male‐specific liver metabolic changes.

In addition to these initially unidentified metabolites in plasma, our analysis identified 24 different free fatty acid species (Table 3). Ten of these were significantly increased in the plasma of 17aE2‐treated males. Only two showed a shared effect in females. 17aE2‐treated female mice show a significant reduction in a different subset of these lipids (Table 3). The effects of 17aE2 on plasma free fatty acid abundance are greatly diminished by castration, with only one of 24 fatty acids changing with treatment (Table 3), consistent with the analysis of castration effects on liver metabolite profile.

**Table 3:** Plasma free fatty acid responses to 17aE2. Significant changes are highlighted in bold

Fatty acid	Effect (p‐value) of 17aE2 treatment within each group				Fold change with 17aE2 treatment			
	Male	Female	Cast male	OVX female	Male	Female	Cast male	OVX female
22:6	**0.0000**	0.18	0.96	**0.05**	1.52	1.08	1.00	1.18
22:3	**0.0002**	**0.01**	0.06	0.44	2.26	1.35	1.49	1.11
22:2	**0.0005**	**0.00**	0.11	0.19	3.57	2.16	1.72	1.30
18:1	**0.001**	0.68	0.36	0.17	1.74	1.07	1.15	1.33
22:4	**0.001**	0.41	0.26	0.86	1.80	1.02	1.24	1.02
20:4	**0.002**	**0.04**	0.69	0.44	1.24	0.91	0.97	1.05
20:1	**0.01**	0.56	0.23	0.48	1.51	1.04	1.12	0.95
18:0	**0.01**	0.59	**0.05**	0.24	1.28	1.01	1.17	1.03
20:3	**0.02**	0.11	0.15	**0.04**	1.53	1.18	1.28	1.22
17:0	**0.02**	0.08	0.99	0.32	1.16	1.07	1.00	1.04
22:0	0.05	0.68	0.95	0.88	1.37	0.96	1.01	1.02
15:0	0.06	0.25	0.44	0.24	1.09	1.05	0.97	1.08
23:0	0.06	0.63	0.33	0.64	1.41	0.91	0.88	0.95
24:0	0.16	0.60	0.28	0.37	1.36	0.89	0.81	0.89
20:1	0.17	0.48	0.38	0.22	1.29	0.92	1.10	0.80
22:0	0.21	0.78	0.86	0.87	1.26	0.98	0.98	0.98
23:0	0.28	0.43	0.27	**0.01**	1.12	0.93	1.07	1.24
16:1	0.46	**0.04**	0.16	0.44	0.90	0.74	0.82	0.90
16:0	0.56	**0.04**	0.14	0.84	1.04	0.93	1.06	1.01
14:0	0.72	**0.01**	0.91	0.13	1.08	0.71	0.99	0.80
18:1	0.74	0.07	0.73	0.81	1.04	0.86	1.03	1.02
24:1	0.77	**0.02**	0.79	0.56	1.06	0.78	0.98	1.09
18:2	0.87	**0.04**	0.41	0.48	0.98	0.83	0.92	0.95
18:3	0.91	**0.04**	0.49	0.15	1.02	0.79	0.89	0.84

### 2.6 Changes in amino acid abundance with 17aE2 are not observed in skeletal muscle

Skeletal muscle is an additional important source of amino acids that can move to the liver during catabolic periods. To test whether the increase in amino acids in livers was correlated with changes in muscle, we assessed whether amino acids levels were increased in quadriceps samples collected at the same time point as livers. We observed no changes in amino acids in response to 17aE2 (Fig. S3). Furthermore, there was a general lack of metabolite responsiveness in muscle to 17aE2, with enrichment analysis failing to show a coordinated response for metabolites in any included pathway (Table S5).

## 3 DISCUSSION

Our results document marked sex‐specific metabolomic effects of 17aE2, a steroid that leads to substantial male‐specific lifespan extension (Strong et al., 2016), in addition to male‐specific improvements in glucose tolerance (Garratt et al., 2017) and lowered age‐related inflammatory dysfunction (Stout et al., 2016). Although 17aE2 has no detectable lifespan effect in females, our data show that it does produce changes in liver and plasma metabolite profiles in this sex, changes that differ from those seen in males. In males, 17aE2 increases the abundance of a variety of amino acids and metabolites involved in the production of urea in the liver. For females, 17aE2 lowers the hepatic abundance of several metabolites associated with betaine metabolism. As with all metabolomic studies, the ability to detect a pathway response is dependent on the method's coverage of related metabolites—there may be metabolomic responses to 17aE2 that our methodological approach did not detect. Nonetheless, our analysis reveals a clear, male‐specific, metabolite response to 17aE2 that is dependent on male gonadal hormone production and could be linked to the health and metabolic benefits of this treatment, although future experimental studies are required to prove a causal link.

The increase in levels of amino acids in 17aE2‐treated males suggests that treated males show changes in aspects of protein metabolism. Elevations of hepatic amino acid levels are also observed with other lifespan extending manipulations in rodents, including calorie restriction (CR) and treatment with acarbose (Gibbs, Brewer, Miyasaki, Patki & Smith, 2017; Green et al., 2017). Acarbose extends lifespan to a much greater degree in male than in female mice (Harrison et al., 2014), while CR works equally in both sexes (Simons, Koch & Verhulst, 2013). A study of fibroblasts from multiple species of rodents, birds, and primates also documented higher levels of 10 different amino acids in fibroblasts derived from longer‐lived species (Ma et al., 2016). Thus, elevated amino acid abundance is a consistent feature of mammalian life extension.

In our study, we also observe elevations in metabolites related to the urea cycle with 17aE2, which are produced as a consequence of amino acid catabolism. This could suggest that 17aE2 promotes the utilization of amino acids as a metabolic fuel, potentially in an amino acid specific way, although this hypothesis requires experimental validation. In our study, we observe significant elevations in glucogenic amino acids (e.g., alanine, arginine, glutamine, and valine), which could suggest that amino acid metabolism provides energy via conversion to glucose. It is notable that valine was significantly increased with 17aE2 treatment while the other branched‐chain amino acids (BCAA, leucine and isoleucine) were not significantly altered. While initiation of catabolism of these three BCAAs occurs via the same enzyme (branched‐chain keto acid dehydrogenase), the subsequent steps are mediated by different enzymes. Furthermore, valine is exclusively glucogenic, while isoleucine is both keto‐ and glucogenic and leucine is exclusively ketogenic. Thus, although the concentration of these amino acids is often highly correlated, their metabolism can differ in a state and tissue‐specific manner (Brosnan & Brosnan 2006). Indeed, while long lifespan has been associated with elevations in selected amino acids in previous studies, the published work has sometimes documented a lack of concordance in BCAA responses. With CR, for example, elevations in leucine are observed in liver without changes in valine or isoleucine (Green et al., 2017), and cells from long‐lived species have higher levels of valine and leucine but not isoleucine (Ma et al., 2016).

Future research is required to test whether the elevations in specific amino acids reflect a net increase in the breakdown of protein and specific amino acids, and whether such effects occur specifically in the liver. We observed no change in amino acid abundance in quadriceps of males treated with 17aE2, indicating that the response observed in the liver is unlikely to reflect an influx of muscle metabolites into the liver, although it is possible that 17aE2 effects on other tissues could be contributing to alterations in liver metabolite profiles. Ultimately, metabolic flux analysis is required to directly test whether the elevations of amino acids represent a net increase in breakdown of existing protein stores or whether alterations in the absorption/synthesis of these compounds are responsible for the observed effects. It would be of further interest to determine whether the observed elevations in amino acids are also observable in unfasted animals, which could suggest differences in basal utilization of different energy substrates.

The elevations in amino acids and urea cycle products with 17aE2 represent a male‐specific phenotype, in that the response is not observed in females. Such sex‐specific effects on a metabolomic scale are consistent with the sex‐specific effects of 17aE2 on glucose tolerance (Garratt et al., 2017) and hypothalamic inflammation (Sadagurski, Cady & Miller, 2017). Furthermore, the response is not observed in males castrated prior to treatment onset, suggesting that this metabolic response to 17aE2 requires the presence of male gonadal hormones. Castrated males show substantially fewer metabolomic responses to 17aE2 in the liver than intact males, intact females or females that were ovariectomised prior to treatment onset. The male‐specific effects of 17aE2 on free fatty acids in plasma are also not observed in castrated males, indicating that the role of male gonadal hormones in mediating responses to 17aE2 is not restricted to the liver, but is also observed in other sites. Experiments where castrated males are treated across life with 17aE2 are now required to determine whether male gonadal hormones are required to elicit the lifespan response to 17aE2. Understanding the hormonal dependence of these responses to 17aE2 will be of importance in the potential translation of this drug, or related steroids, for improving late‐life health outcomes in humans of both sexes (Gonzalez‐Freire, Diaz‐Ruiz & de Cabo, 2017).

Future experiments are required to establish why the metabolic effects of 17aE2 are dependent on the presence of male gonadal hormones and the specific steroids that underlie sexually dimorphic responses to 17aE2. The major hormone altered by male castration is testosterone, and therefore, it is likely that the effectiveness of 17aE2 on mouse metabolism is dependent on circulating testosterone. While direct manipulation of testosterone levels over extended periods of life in mice is technically unfeasible (due to the pulsatile nature of its release and changes in concentration with age), experiments where expression of the androgen receptor (AR) is manipulated genetically may provide an insight into the role of androgenic signaling in generating treatment responses to 17aE2, and the role of androgenic signaling in aging more generally. The apparent dependence of 17aE2 treatment responses on male gonadal hormones could occur if 17aE2 inhibits or protects against a downstream action of testosterone, which ultimately constrains the male‐specific metabolomic response. As 17aE2 is a 5 alpha reductase inhibitor (Schriefers et al., 1991), one such mechanism might involve inhibition of testosterone's conversion to dihydrotestosterone, which is a more potent binder of the AR. This could inhibit specific effects of testosterone on metabolism, which include protein anabolism and inhibition of urea cycling (Lam et al., 2017; Rossetti, Steiner & Gordon, 2017), and contribute to the observed elevation of amino acids and urea cycling. Under this hypothesis, inhibition AR expression in mice would be expected to inhibit 17aE2 responses, and other 5‐alpha reductase inhibitors might induce similar effects to 17aE2.

Another related hypothesis is that the activity, metabolism and/or signaling effects of 17aE2 are dependent on testosterone, and without the presence of testosterone the biological effects of 17aE2 are weaker. Two of the liver metabolites that showed strong, sex‐specific responses to 17aE2 were the estrogenic compounds estriol‐3‐sulfate and 16‐oxoestradiol 3‐sulfate—products that can be generated from metabolism of estradiol. Estriol is produced from the placenta during pregnancy in females and during nonpregnant states is generated from estradiol or estrone via 16‐alpha hydroxylation (Longcope 1984). Most estriol in circulation is conjugated, of which estriol‐3‐sulfate is the most abundant form (Tanaka et al. 1984). 16‐oxoestradiol is an intermediate metabolite in the conversion of estradiol to estriol (Pasqualini & Kincl 1984). Considering that many of the xenobiotic enzymes that metabolize steroids show sexual dimorphism in expression (Waxman & Holloway, 2009), it is possible that 17aE2 may be metabolized into a different form, in either males or females, which could influence either the transport of this steroid, its activation/inactivation, or its ability to bind to estrogen receptors. 17aE2 is readily conjugated into various forms in humans (Hobe et al. 2002), and understanding the metabolism of this steroid may be important when considering translational potential of this drug to humans.

The longevity benefits of 17aE2 highlight the potentially important role of steroids in control of mammalian lifespan, and a greater understanding of changes in steroidogenic signaling that occur with life extension could provide an insight into pathways and molecules that control aging in one or both sexes. In relation to circulating estrogens, much previous research focus has been placed on the role of 17‐β estradiol in controlling metabolism and generating sex differences in health and metabolic dysfunction at different periods of life. However, the role of 17aE2 in slowing mouse aging, improving mouse metabolism, and generating metabolomic responses associated with life‐extension points to an important role of additional estrogens in controlling mammalian health and aging. 17aE2 has a weaker binding affinity to classical estrogen receptors than 17‐β estradiol (Anstead, Carlson & Katzenellenbogen, 1997), although experiments with estrogen receptor knockout mice are required to fully establish whether 17aE2 acts independently or through these receptors to induce its metabolic and anti‐aging effects. Recent research suggests the metabolic benefits of 17‐β estradiol on mouse weight and body composition can also be at least partly recapitulated by treatment with chemically altered, pathway preferential estrogens (PaPEs). The structural modifications of PaPEs cause these molecules to have a weaker binding affinity to estrogen receptors, and so do not cause feminization, but are still able to activate the extranuclear‐initiated ER signaling pathway and activate metabolic processes sufficient to influence fat deposition (Madak‐Erdogan et al., 2016). Future experiments with PaPEs and more diverse estrogens like estriol (which was observed to be strongly elevated by 17aE2 treatment) could provide a greater insight into the role of different aspects of estrogenic signaling in metabolism and aging, and might ultimately lead to interventions that can be applied to protect against age‐related disease and metabolic dysfunction in humans.

## 4 EXPERIMENTAL PROCEDURES

UM‐HET3 mice were produced as previously described (Miller et al., 2014; Strong et al., 2008). The mothers of the test mice were CByB6F1/J, JAX stock #100009, whose female parents are BALB/cByJ and whose male parents are C57BL/6J. The fathers of the test mice were C3D2F1/J, JAX stock #100004, whose mothers are C3H/HeJ, and whose fathers are DBA/2J. Mice in breeding cages received Purina 5008 mouse chow, and weaned animals were fed Purina 5LG6.

Mice were housed as previously described (Miller et al., 2014; Strong et al., 2008) in plastic cages with metal tops, using ¼‐inch corn‐cob bedding (Bed O'Cobs, produced by The Andersons, PO Box 114, Maumee, OH, USA). Mice were given free access to water, using water bottles rather than an automated watering system. Mice were housed in ventilated cages and were transferred to fresh cages every 14 days. Temperature was maintained within the range of 21–23°C.

### 4.1 Surgical procedures

At three months of age, all animals went through castration, ovariectomy, or a sham procedure. All animals were anaesthetized by injection of 250 mg/kg tribromoethanol, and given a single preoperative injection of the analgesia carprofen, at 5 mg/kg.

### 4.2 Castration and sham castration

After surgical preparation, an incision was made in the caudal end of each scrotal sac, the testicle was pulled through the incision by gentle traction, and the blood vessels, vas deferens, and deferential vessels were clamped and sutured. The incision was closed with tissue adhesive. For sham surgery, the testicles were exteriorized and then replaced in the scrotum, without being ligated or excised.

### 4.3 Ovariectomy or sham ovariectomy

After surgical preparation, an incision was made on the left side perpendicular to the vertebral column approximately midway between the iliac crest and the last rib. The ovarian fat pad was grasped and exteriorized. The pedicle under the ovarian blood vessels and fat pad under the ovary were grasped and crushed, the pedicle cut on the ovary side and the ovary removed, and the blood vessels tied with absorbable suture. The abdominal wall was closed with absorbable suture, and skin was closed with staples. The procedure was then repeated on the opposite side. For sham ovariectomy, animals underwent the same surgical procedure, but the ovary and fat pad were exteriorized and replaced without being excised.

### 4.4 Diets

At four months of age, animals in different sibling groups were randomly allocated to control or 17aE2 treatment. Animals in the control group remained on the 5LG6 diet, while animals allocated to 17aE2 had their diet switched to a food containing this drug.

Diets were prepared by TestDiet, Inc., a division of Purina Mills (Richmond, IN, USA). Purina 5LG6 food contained 17aE2 and was also used as the control diet. 17aE2 was purchased from Steraloids Inc. (Newport, RI, USA) and mixed at a dose of 14.4 milligrams per kilogram diet (14.4 ppm). These methods followed those used by the NIA Interventions Testing Program.

### 4.5 Metabolomics analysis

Livers, muscle, and plasma were harvested during the morning, from 12‐month‐old mice after 18 hr of fasting. Samples were frozen with liquid nitrogen and stored at −70°C. Tissues were ground under liquid nitrogen prior to metabolomics analysis. See Supplementary methods for muscle metabolomic analysis protocol.

### 4.6 Sample preparation

Mouse liver samples were analyzed by liquid chromatography–mass spectrometry (LC‐MS). Snap‐frozen and pulverized liver samples were resuspended in cold 80:20 methanol:water at 40 μg/μl. Samples were subjected to three freeze–thaw cycles, with alternating 37°C and −80°C liquid baths in 30‐s intervals to ensure complete cell lysis, vortexing, and a 2‐min sonication. Samples were thereafter placed in the −20°C freezer for 30 min to allow for precipitation of protein, vortexed for 60 s, and centrifuged at 14,000 g × 10 min at 4°C. Supernatants were collected and transferred to LC‐MS vials. For measurement of small lipids, identical preparation protocols were used with the exception of 80:20 ethanol:water for extraction solvent, followed by solid‐phase extraction of lipid metabolites using a Strata‐X polymeric 10 mg/ml 96‐well SPE plate, as previously described (Watrous et al., 2017).

### 4.7 LC‐MS‐based metabolomics

LC‐MS/MS‐based metabolomics analysis was performed using a Thermo QExactive orbitrap mass spectrometer coupled to a Thermo Vanquish UPLC system. For analysis of polar molecules, we achieved chromatographic separation using a Millipore (Sequant) Zic‐pHILIC 2.1 × 150 mm 5um column maintained at 25°C. Compounds were eluted via a 19‐min linear gradient starting from 90:10 acetonitrile:20 mm ammonium bicarbonate to 45:55 acetonitrile:20 mM ammonium bicarbonate. For chromatographic separation of bioactive lipids, we used a Phenomenex Kinetex C18 (1.7 μm particle size, 100 × 2.1 mm) column maintained at 50⁰C using mobile phases A (70% water, 30% acetonitrile and 0.1% acetic acid) and B (50% acetonitrile, 50% isopropanol, 0.02% acetic acid) running the following gradient: 1% B from −1.00 to 0.25 min, 1% to 55% B from 0.25 to 5.00 min, 55% to 99% B from 5.00 to 5.50 min, and 99% B from 5.50 to 7.00 min. For the analysis of nonpolar lipids, we used the same column and column temperature as for the bioactive lipids analysis, but separation in positive ion mode was achieved using mobile phases A (Water with 0.1% formic acid and 10 mM ammonium formate) and B (50% acetonitrile, 50% isopropanol, and 0.1% formic acid) running at 5% B from −2.00 to 0.25 min, 5% to 50% B from 0.25 to 2.00 min, 50% to 99% B from 5.00 to 9.50 min, and 99% B from 9.50 to 13.00 min. For separation in negative ion mode mobile phase A (1 mM ammonium fluoride) and B (50% acetonitrile, 50% isopropanol, and 1 mM ammonium fluoride) were run with the same gradient as just described. For all methods, a Thermo QExactive orbitrap mass spectrometer was operated in positive and negative ion modes for nonpolar lipids and polar molecules and negative mode only for bioactive lipid analysis.

### 4.8 Chemical networking and metabolite identification

Collected data were imported into the mzMine 2.26 software suite for analysis. Pure standards were used for identification of metabolites through manual inspection of spectral peaks and matching of retention time and MS1 accurate mass, with confirmation of identification through comparison to MS/MS fragmentation patterns. For unknown metabolites, chemical networking was performed by Global Natural Products Social molecular networking (GNPS, http://gnps.ucsd.edu) and all visualization was performed in Cytoscape (Watrous et al., 2012). The data were clustered with a parent mass tolerance of 1.0 Da and a MS/MS fragment ion tolerance of 0.15 Da. The spectra in the network were searched against GNPS spectral libraries, and all connections were required to a minimum cosine score of 0.7 and at least 3 matched peaks. Estriol 3‐sulfate and 16‐oxoestradiol 3‐sulfate were identified through manual inspection of spectral peaks with confirmation of identification through comparison to MS/MS fragmentation patterns.

### 4.9 Statistical analysis

#### 4.9.1 Analysis of untargeted metabolites

We first focused on metabolite responses that occur in mice where gonadal hormone production was unaltered, that is, mice that had only been through sham surgery, as data on lifespan responses to 17aE2 were conducted in male and female mice without surgical manipulation (Strong et al., 2016).

For each metabolite, we fitted a two‐way ANOVA model testing the effects of sex, treatment, and the interaction between sex and treatment with data from sham‐operated males and females. The interaction term in the model was our primary interest as it indicates that the effect of 17aE2 treatment was modified by sex. By scanning through all interactions, we were able to identify metabolites that reveal significant treatment by sex interaction. Because multiple two‐way interaction effects were tested across the dataset, we adjusted raw *p*‐values with False Discovery Rate (FDR) method (Benjamini & Hochberg, 1995).

For those metabolites that showed a significant sex by treatment interaction, we subsequently tested whether male castration or female ovariectomy influenced the treatment response within either sex. In this analysis, data were split according to sex, then tested for an effect of surgery (e.g., within males, castration or sham castration; within females, ovariectomy or sham ovariectomy), treatment, and an interaction between surgery and treatment. An interaction between surgery and treatment indicates that the response to 17aE2 depends on whether animals had been gonadectomized (and gonadal hormones are removed) or not.

#### 4.9.2 Analysis of known metabolites

For analysis of metabolites that were identified against a standard reference library, we conducted Quantitative Metabolomic Set Enrichment Analysis using Metaboanalyst 3.0 (Xia & Wishart, 2010) after matching metabolites to their respective HMDB IDs. Data for control and 17aE2 animals within each sex and surgery group were uploaded to the metaboanalyst 3.0 website separately. We conducted quantitative enrichment analysis by uploading a concentration table that showed abundance values for each metabolite for each individual, with animals differentiated according to whether they were control or 17aE2. Data were log‐transformed, and then, enrichment analysis was conducted against the “pathway‐associated metabolite sets” library. Enrichment for a specific pathway was considered statistically significant when the FDR *p*‐value was lower than 0.05.

We also looked within the known metabolite data to test whether there were changes in individual metabolites that differed depending on sex, and whether sex‐specific responses in these instances were altered by surgical removal of gonads in either males or females. These statistical tests were conducted as part of the larger analysis of untargeted metabolite responses, but we present sex by treatment interaction responses that are uncorrected for FDR.

## CONFLICT OF INTEREST

None declared.
