# Supplementary material for: Male lifespan extension with 17‐α estradiol is linked to a sex‐specific metabolomic response modulated by gonadal hormones in mice
Source: Aging Cell. 2018 May 27;17(4):e12786. doi: 10.1111/acel.12786 (PMC6052402; doi:10.1111/acel.12786)
Supplement: Supplementary file 1 [file ACEL-17-na-s001.docx]

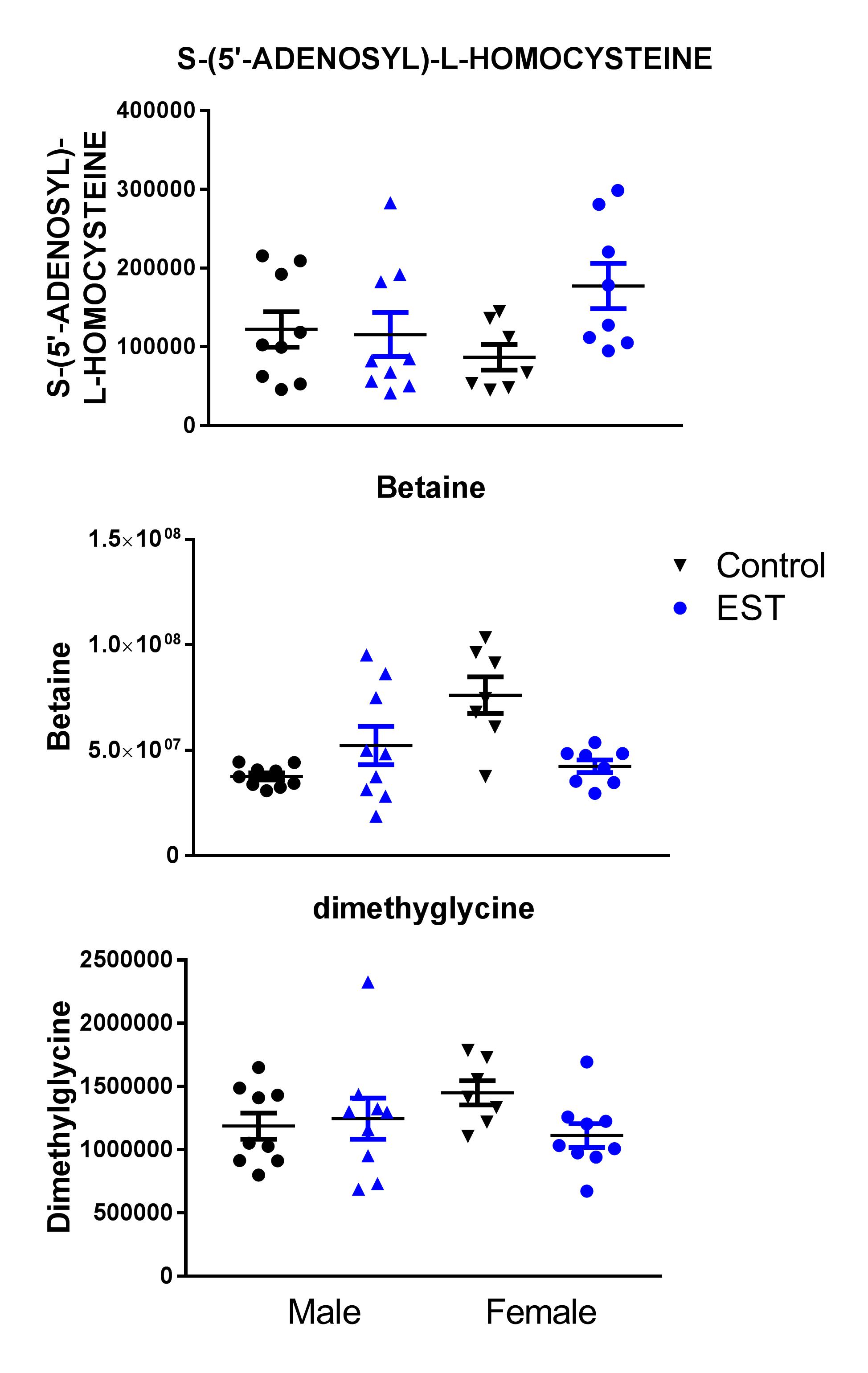


Figure S1. Metabolites associated with “Betaine metabolism” changing in females with 17aE2 treatment.


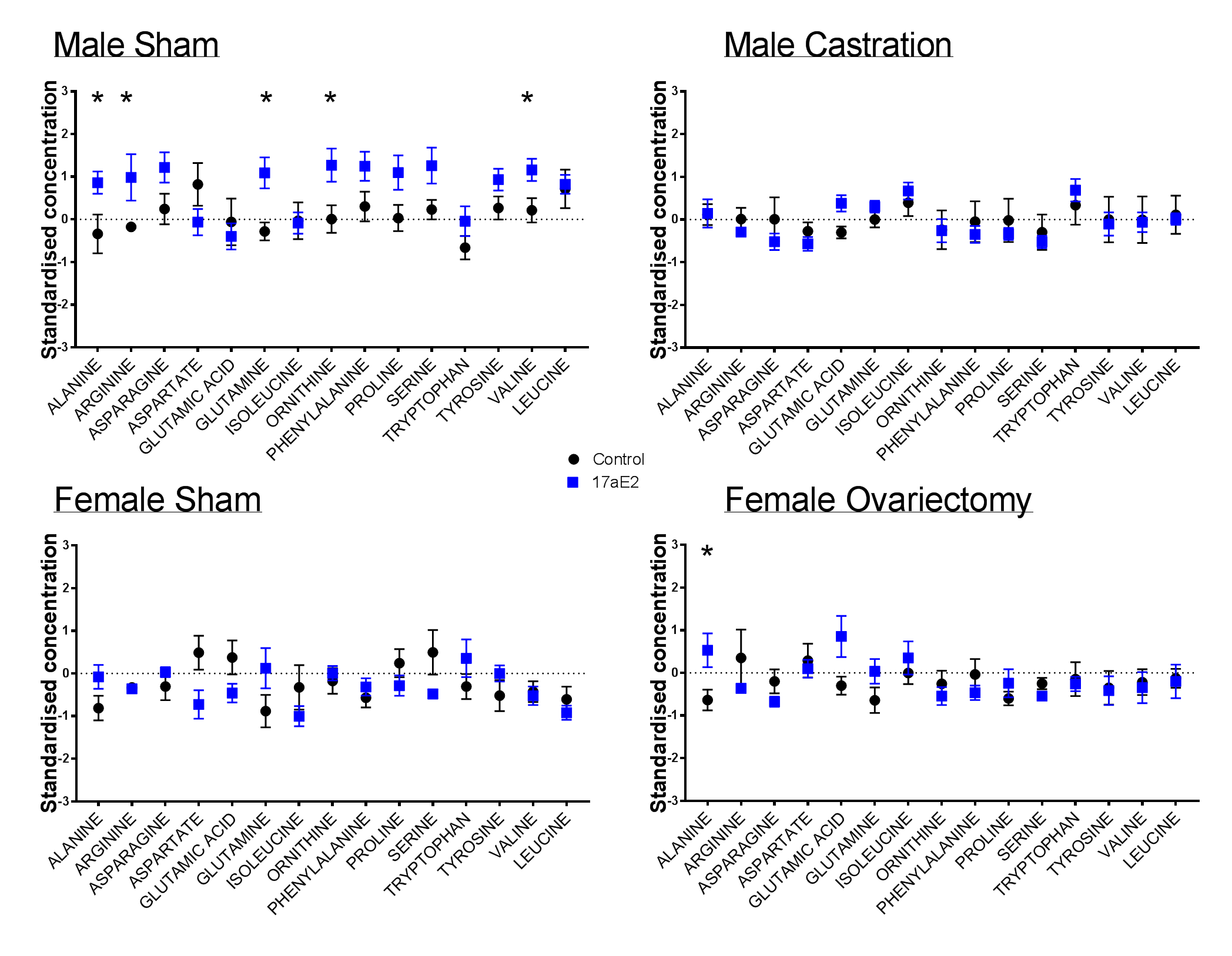


Figure S2. The male-specific metabolomic response to 17aE2 is ablated in castrated males.

Figure S2. Amino acid abundance is unaffected by 17aE2 in muscle. N= 7 per treatment group. See supplementary methods for method.

Table S1. Metabolites that are significantly affected by 17aE2 treatment in females. Arrows indicate direction of change with 17aE2 treatment. P values are generated by a Student’s T-test, The comparable analysis for each metabolite is also shown for males, castrated males and OVX females. Sample sizes are shown in Table 2.

Table S2. Pathways associated with metabolite responses to 17aE2 treatment in male and female mice.

| **Pathway** | **Compounds in pathway** | **Hits** | **Statistic Q** | **Expected Q** | **Raw p** | **FDR p** |
| --- | --- | --- | --- | --- | --- | --- |
| **Male** |  |  |  |  |  |  |
| UREA CYCLE | 20 | 9 | 33.63 | 6.67 | 0.0003 | 0.0083 |
| PROTEIN BIOSYNTHESIS | 19 | 13 | 35.12 | 6.67 | 0.0003 | 0.0083 |
| ALPHA LINOLENIC ACID AND LINOLEIC ACID METABOLISM | 9 | 6 | 19.47 | 6.67 | 0.0166 | 0.2098 |
| SELENOAMINO ACID METABOLISM | 15 | 1 | 32.59 | 6.67 | 0.0209 | 0.2098 |
| ARACHIDONIC ACID METABOLISM | 37 | 1 | 31.17 | 6.67 | 0.0246 | 0.2098 |
| ARGININE AND PROLINE METABOLISM | 26 | 9 | 18.34 | 6.67 | 0.0414 | 0.2098 |
| STEROIDOGENESIS | 32 | 2 | 23.99 | 6.67 | 0.0478 | 0.2098 |
| BETA-ALANINE METABOLISM | 13 | 6 | 16.03 | 6.67 | 0.0542 | 0.2098 |
|  |  |  |  |  |  |  |
| **Female** |  |  |  |  |  |  |
| BETAINE METABOLISM | 10 | 4 | 44.94 | 7.14 | 0.0006 | 0.0322 |
| METHIONINE METABOLISM | 24 | 7 | 24.15 | 7.14 | 0.0123 | 0.2927 |
| PROTEIN BIOSYNTHESIS | 19 | 11 | 17.64 | 7.14 | 0.0185 | 0.2927 |
| MALATE-ASPARTATE SHUTTLE | 8 | 2 | 28.23 | 7.14 | 0.0233 | 0.2927 |
| INTRACELLULAR SIGNALLING THROUGH ADENOSINE RECEPTORs AND ADENOSINE | 7 | 1 | 29.97 | 7.14 | 0.0347 | 0.2927 |
| ALPHA LINOLENIC ACID AND LINOLEIC ACID METABOLISM | 9 | 6 | 20.30 | 7.14 | 0.0382 | 0.2927 |
| UREA CYCLE | 20 | 6 | 17.55 | 7.14 | 0.0430 | 0.2927 |
| ASPARTATE METABOLISM | 12 | 6 | 16.88 | 7.14 | 0.0434 | 0.2927 |

| **Pathway** | **Compounds in pathway** | **Hits** | **Statistic Q** | **Expected Q** | **Raw p** | **FDR p** |
| --- | --- | --- | --- | --- | --- | --- |
| **Castrated Male** |  |  |  |  |  |  |
| GLUTAMATE METABOLISM | 18 | 5 | 30.94 | 7.14 | 0.0023 | 0.1251 |
| VITAMIN B6 METABOLISM | 10 | 1 | 44.56 | 7.14 | 0.0065 | 0.1800 |
| STEROIDOGENESIS | 32 | 2 | 37.79 | 7.14 | 0.0106 | 0.1952 |
| ALANINE METABOLISM | 6 | 2 | 24.42 | 7.14 | 0.0246 | 0.2708 |
| GLUCOSE-ALANINE CYCLE | 12 | 2 | 24.42 | 7.14 | 0.0246 | 0.2708 |
| GLUTAMATE METABOLISM | 18 | 5 | 30.94 | 7.14 | 0.0023 | 0.1251 |
| VITAMIN B6 METABOLISM | 10 | 1 | 44.56 | 7.14 | 0.0065 | 0.1800 |
| STEROIDOGENESIS | 32 | 2 | 37.79 | 7.14 | 0.0106 | 0.1952 |
|  |  |  |  |  |  |  |
| **Ovariectomized Female** |  |  |  |  |  |  |
| INTRACELLULAR SIGNALLING THROUGH ADENOSINE RECEPTORs AND ADENOSINE | 7 | 1 | 50.21 | 6.67 | 0.0021 | 0.0619 |
| GLUTAMATE METABOLISM | 18 | 5 | 27.91 | 6.67 | 0.0023 | 0.0619 |
| ALPHA LINOLENIC ACID AND LINOLEIC ACID METABOLISM | 9 | 6 | 29.04 | 6.67 | 0.0056 | 0.0660 |
| BETAINE METABOLISM | 10 | 4 | 28.25 | 6.67 | 0.0067 | 0.0660 |
| METHIONINE METABOLISM | 24 | 7 | 19.77 | 6.67 | 0.0078 | 0.0660 |
| ALANINE METABOLISM | 6 | 2 | 35.39 | 6.67 | 0.0084 | 0.0660 |
| GLUCOSE-ALANINE CYCLE | 12 | 2 | 35.39 | 6.67 | 0.0084 | 0.0660 |
| HISTIDINE METABOLISM | 11 | 3 | 24.60 | 6.67 | 0.0156 | 0.1074 |

Table **S3**. Pathways associated with metabolite responses to 17aE2 treatment in castrated males and OVX females.

Table S4. Metabolites showing a male-specific response to 17aE2 treatment in plasma. Data is split into in relation whether the male response represents an increase or a decrease.

| Metabolite | Treatment interaction  (P-value 2 – Way ANOVA) | | | Effect of 17aE2  (P-Value Student’s T-Test) | | | |
| --- | --- | --- | --- | --- | --- | --- | --- |
|  | Sex | Castration | OVX | Male | Female | Cast male | OVX female |
|  | (intact mice) | (male) | (female) |  |  |  |  |
| Increasing in males |  |  |  |  |  |  |  |
| P0001313 | 0.0001 | 0.015 | 0.53 | 0.00015 | 0.67 | 0.43 | 0.65 |
| P0001071 | 0.0002 | 0.052 | 0.059 | 0.00020 | 0.50 | 0.54 | 0.046 |
| P0004550 | 0.0003 | 0.20 | 0.089 | 0.00002 | 0.31 | 0.16 | 0.0036 |
| P0000898 | 0.0003 | 0.040 | 0.16 | 0.00001 | 0.98 | 0.46 | 0.063 |
| P0001362 | 0.0007 | 0.019 | 0.14 | 0.00013 | 0.50 | 0.010 | 0.067 |
| P0004536 | 0.0011 | 0.36 | 0.056 | 0.00028 | 0.83 | 0.25 | 0.017 |
| FAHFA_631.472635 | 0.0003 | 0.15 | 0.19 | 0.00013 | 0.31 | 0.435 | 0.38 |
| FAHFA_607.472635 | 0.0008 | 0.030 | 0.10 | 0.00065 | 0.24 | 0.83 | 0.26 |
| Decreasing in males |  |  |  |  |  |  |  |
| P0001224 | 0.0001 | 0.003 | 0.007 | 0.00009 | 0.22 | 0.98 | 0.011 |
| P0002303 | 0.0004 | <0.001 | 0.014 | 0.00000 | 0.28 | 0.052 | 0.017 |
| P0001913 | 0.0007 | 0.001 | 0.11 | 0.00028 | 0.15 | 0.21 | 0.012 |
| P0002636 | 0.0012 | 0.002 | 0.11 | 0.00020 | 0.089 | 0.67 | 0.067 |
| P0002637 | 0.0002 | 0.005 | 0.048 | 0.00040 | 0.0051 | 0.61 | 0.056 |

Table S5. No changes in enrichment for metabolites associated with specific pathways with 17aE2 treatment in intact male quadricep muscle. The first 10 null-results are shown for reference.

| **Pathway** | **Compounds in pathway** | **Hits** | **Statistic Q** | **Expected Q** | **Raw p** | **FDR p** |
| --- | --- | --- | --- | --- | --- | --- |
| Thyroid hormone synthesis | 13 | 1 | 20.587 | 7.1429 | 0.089364 | 0.95864 |
| Plasmalogen Synthesis | 26 | 2 | 15.634 | 7.1429 | 0.11509 | 0.95864 |
| Phosphatidylinositol Phosphate Metabolism | 17 | 1 | 17.778 | 7.1429 | 0.11749 | 0.95864 |
| Cardiolipin Biosynthesis | 11 | 2 | 12.687 | 7.1429 | 0.17057 | 0.95864 |
| Biotin Metabolism | 8 | 2 | 10.587 | 7.1429 | 0.22566 | 0.95864 |
| Catecholamine Biosynthesis | 20 | 2 | 10.318 | 7.1429 | 0.23436 | 0.95864 |
| Sulfate/Sulfite Metabolism | 22 | 1 | 10.141 | 7.1429 | 0.24736 | 0.95864 |
| Ubiquinone Biosynthesis | 20 | 1 | 10.141 | 7.1429 | 0.24736 | 0.95864 |
| Fructose and Mannose Degradation | 32 | 4 | 8.7705 | 7.1429 | 0.27953 | 0.95864 |
| Spermidine and Spermine Biosynthesis | 18 | 3 | 8.9281 | 7.1429 | 0.28232 | 0.95864 |

**Supplementary methods**

Muscle metabolomics data were acquired at the NIH West Coast Metabolomics Center, UC Davis, using an untargeted analysis for primary metabolites.

**Instruments:**

Gerstel CIS4 –with dual MPS Injector/

Agilent 6890 GC-

Pegasus III TOF MS

**Injector conditions:**

Agilent 6890 GC is equipped with a Gerstel automatic liner exchange system (ALEX) that includes a multipurpose sample (MPS2) dual rail, and a Gerstel CIS cold injection system (Gerstel, Muehlheim, Germany) with temperature program as follows: 50°C to 275°C final temperature at a rate of 12 °C/s and hold for 3 minutes. Injection volume is 0.5 μl with 10 μl/s injection speed on a splitless injector with purge time of 25 seconds. Liner (Gerstel #011711-010-00) is changed after every 10 samples, (using the Maestro1 Gerstel software vs. 1.1.4.18). Before and after each injection, the 10 μl injection syringe is washed three times with 10 μl ethyl acetate.

**Gas Chromatography conditions:**

A 30 m long, 0.25 mm i.d. Rtx-5Sil MS column (0.25 μm 95% dimethyl 5% diphenyl polysiloxane film) with additional 10 m integrated guard column is used (Restek, Bellefonte PA). 99.9999% pure Helium with built-in purifier (Airgas, Radnor PA) is set at constant flow of 1 ml/min. The oven temperature is held constant at 50°C for 1 min and then ramped at 20°C/min to 330°C at which it is held constant for 5 min.

**Mass spectrometer settings:**

A Leco Pegasus IV time of flight mass spectrometer is controlled by the Leco ChromaTOF software vs. 2.32 (St. Joseph, MI). The transfer line temperature between gas chromatograph and mass spectrometer is set to 280°C. Electron impact ionization at 70V is employed with an ion source temperature of 250°C. Acquisition rate is 17 spectra/second, with a scan mass range of 85-500 Da.
